# The cellular heat shock response monitored by chemical exchange saturation transfer MRI

**DOI:** 10.1038/s41598-020-68022-1

**Published:** 2020-07-06

**Authors:** Dennis Kleimaier, Steffen Goerke, Cordula Nies, Moritz Zaiss, Patrick Kunz, Peter Bachert, Mark E. Ladd, Eric Gottwald, Lothar R. Schad

**Affiliations:** 1 Computer Assisted Clinical Medicine, Heidelberg University, Theodor-Kutzer-Ufer 1-3, 68167 Mannheim, Germany; 2 Division of Medical Physics in Radiology, German Cancer Research Center (DKFZ), Heidelberg, Germany; 3 Institute of Functional Interfaces, Karlsruhe Institute of Technology, Karlsruhe, Germany; 4 Neuroradiology, University of Erlangen-Nürnberg, Erlangen, Germany; 5 Division of Functional Genome Analysis, German Cancer Research Center (DKFZ), Heidelberg, Germany; 6 Faculty of Physics and Astronomy, Heidelberg University, Heidelberg, Germany; 7 Faculty of Medicine, Heidelberg University, Heidelberg, Germany

**Keywords:** Magnetic resonance imaging, Molecular imaging

## Abstract

CEST-MRI of the rNOE signal has been demonstrated in vitro to be closely linked to the protein conformational state. As the detectability of denaturation and aggregation processes on a physiologically relevant scale in living organisms has yet to be verified, the aim of this study was to perform heat-shock experiments with living cells to monitor the cellular heat-shock response of the rNOE CEST signal. Cancer cells (HepG2) were dynamically investigated after a mild, non-lethal heat-shock of 42 °C for 20 min using an MR-compatible bioreactor system at 9.4 T. Reliable and fast high-resolution CEST imaging was realized by a relaxation-compensated 2-point contrast metric. After the heat-shock, a substantial decrease of the rNOE CEST signal by 8.0 ± 0.4% followed by a steady signal recovery within a time of 99.1 ± 1.3 min was observed in two independent trials. This continuous signal recovery is in coherence with chaperone-induced refolding of heat-shock induced protein aggregates. We demonstrated that protein denaturation processes influence the CEST-MRI signal on a physiologically relevant scale. Thus, the protein folding state is, along with concentration changes, a relevant physiological parameter for the interpretation of CEST signal changes in diseases that are associated with pathological changes in protein expression, like cancer and neurodegenerative diseases.

## Introduction

Chemical exchange saturation transfer (CEST) MRI combines the spectroscopic information from low concentrated organic compounds, such as proteins^1,2^ and metabolites^3–5^, with high-resolution imaging comparable to normal water MRI^6,7^. The relayed nuclear Overhauser effect (rNOE) along with the amide proton transfer CEST signal has been shown to provide valuable information for various neuro-oncological clinical questions^8–10^, such as the differentiation of histologic and genetic subtypes of glioma^9^ or treatment response assessment^11^.

The rNOE signal is a protein-based magnetization transfer which depends on the protein content^12,13^, and is insensitive to pH^12,14^. In addition, the rNOE signal is closely linked to the protein conformational state^15–18^. Upon selective protein unfolding of in vitro model solutions, the rNOE signal decreased in accordance with the protein folding state^17–19^. The study of protein aggregation of Amyloid ß (Aß peptide), Huntingtin protein and cell lysates also resulted in a reduction of the rNOE signal^16^. Therefore, this protein-based magnetization transfer signal has great potential for the diagnostic imaging of diseases associated with pathological changes in protein expression and proteome structure, like cancer or neurodegenerative diseases. An initial investigation of Alzheimer’s disease in amyloid precursor protein transgenic mice^15^ revealed a reduction in the aliphatic rNOE signal, which was attributed to aggregated proteins. Nonetheless, the detectability of denaturation processes on a physiologically relevant scale in living organisms by CEST MRI has yet to be verified experimentally.

To demonstrate the detectability of protein denaturation processes and the subsequent cellular heat shock response using CEST MRI, a mild non-lethal heat shock was applied to living cells in this study. The biological function of proteins is linked to their 3D structural integrity. However, a slight elevation in temperature above the optimal growth temperature of cells causes the denaturation of certain proteins which are highly prone to aggregation and loss of function. To mitigate the toxic effects of misfolded proteins caused by a heat shock, cells transiently overexpress molecular chaperones^20,21^. They prevent protein aggregation during heat shock as well as dissolve and refold protein aggregates upon return to native temperatures^20,21^. Based on previous MR studies on protein denaturation^16–18^, application of a mild, non-lethal cellular heat shock, which causes the accumulation of misfolded proteins, and the subsequent chaperone-induced refolding of misfolded proteins is expected to affect the protein-based rNOE signal. Therefore, the goal of the current study was to non-invasively monitor the cellular heat shock response of living cells in an MR-compatible bioreactor system using the aliphatic rNOE signal.

Gottwald et al.^22^ recently proposed an MR-compatible bioreactor system for the non-invasive investigation of living cells by MRI. This bioreactor system allows for the study of an organotypic 3D cell culture cultivated on a collagenized microcavity array (MCA) in a precisely adjustable environment^23,24^. An external perfusion pump provides a homogenous, continuous flow of medium to supply the 3D cell culture with nutrients and oxygen^22^. Investigations based on X-Nuclei MRI^25–27^, i.e. sodium triple-quantum signal^25,28^, showed the possibility to detect the cellular response to Na^+^/K^+^-ATPase blockage^24^ and ischemia^23^ in this bioreactor system.

In this study, the detectability of protein denaturation processes by CEST MRI was verified by application of a mild, non-lethal heat shock of 42 °C for 20 min to living cells using such an MR-compatible bioreactor at 9.4 T. The origin of CEST signals in this bioreactor system was determined by evaluation of the individual contribution of CEST signals from medium, collagenized MCAs and cells to the total Z-spectrum. For the monitoring of the cellular heat shock response, a reliable and fast high-resolution CEST imaging of the rNOE signal was implemented. In doing so, the applicability of a recently proposed 2-point contrast metric^15^ to this bioreactor system was investigated to perform fast dynamic CEST measurements during the heat shock experiments. To exclude confounding changes in the rNOE signal during dynamic measurements caused by a protein denaturation of medium and collagenized MCAs or due to temporal signal fluctuations, two additional dynamic control experiments were performed. To verify the detectability of the cellular heat shock response by the rNOE CEST signal, two independent 3D cell cultures were investigated.

## Results

### Contributions to the Z-spectrum

**Figure 1:**
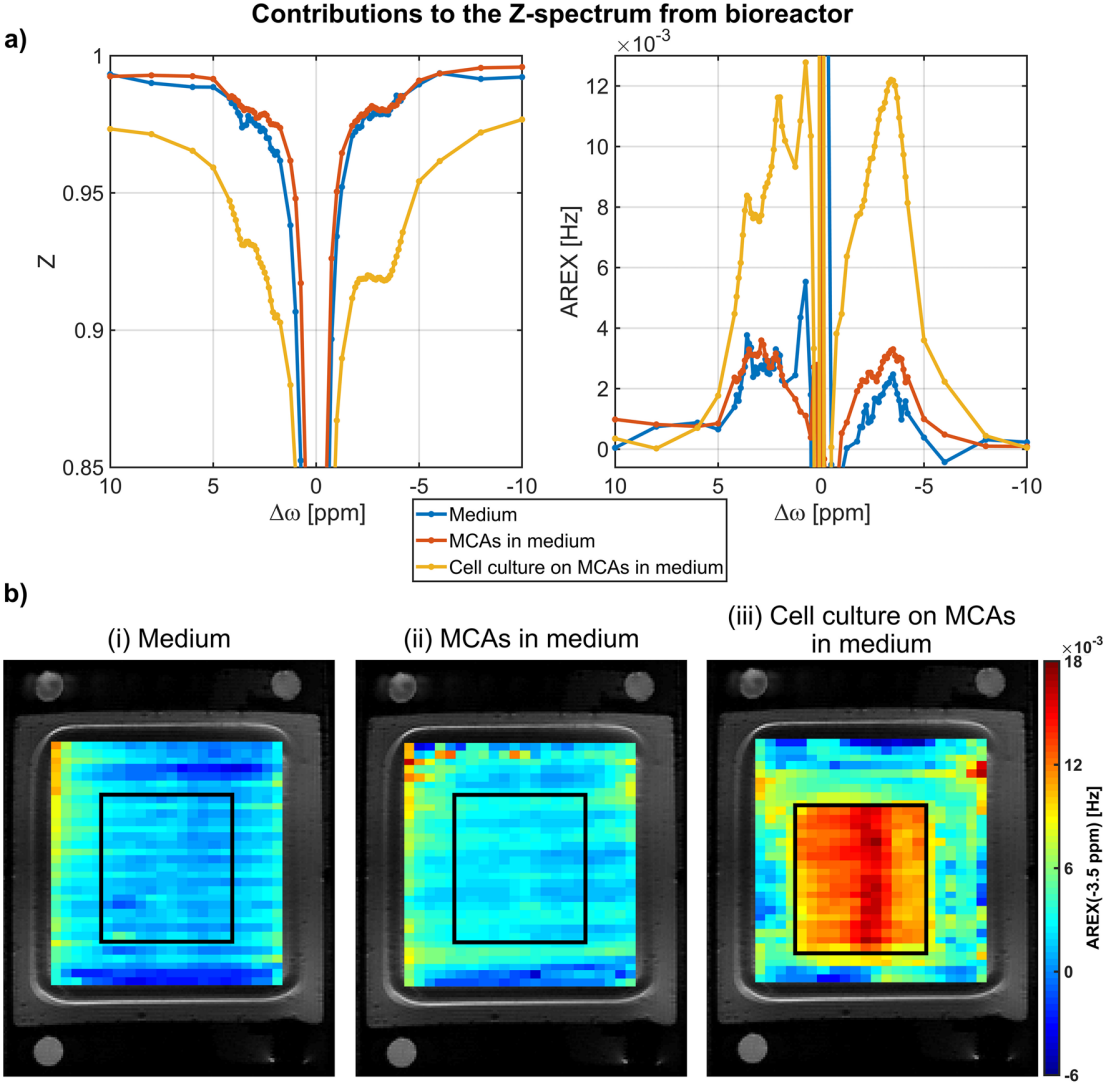
(**a**) Contribution of CEST signals from medium, collagenized MCAs and cells to the total Z-spectrum and AREX spectrum (B_0_ = 9.4 T and B_1_ = 0.8 µT). The CEST signals mainly originated from the 3D cell culture of 16–18·10^6^ HepG2 cells, while proteins in medium and collagenized MCAs contributed only to a small background signal (Table 1). (**b**) rNOE AREX images of the different contributions to the −3.5 ppm rNOE signal overlaid on a ^1^H RARE image. In the two images without cells, a small rNOE signal across the bioreactor was observed, while the presence of cells led to distinct rNOE signals in the expected size and location of the cavity structure on the MCAs.

Three possible contributors to the Z-spectrum of the bioreactor have been identified: (I) The medium containing amino acids, metabolites, and proteins; (II) The MCAs which were collagenized to provide an extracellular matrix for the attachment of the cells and (III) a 3D cell culture located on the MCAs. The contributions of the medium, two collagenized MCAs in medium and a cell culture on the collagenized MCAs in medium to the Z-spectrum are shown in Fig. 1a. For medium and collagenized MCAs only a small contribution to the Z-spectrum was observed, while the presence of a 3D cell culture led to distinctive CEST signals of amide, amine and guanidinium protons resonating around 3.5, 2.7 and 2.0 ppm. The aliphatic rNOE signal at around −3.5 ppm evaluated by the apparent exchange-dependent relaxation (AREX)^29^ metric was also larger by more than a factor of three compared to the rNOE signal from collagenized MCAs in medium (Table 1). Therefore, the CEST signals mainly originated from the 3D cell culture, while medium and collagenized MCAs contributed only to a small background signal which is also visible in the −3.5 ppm AREX images of the three contributions (Fig. 1b).

**Table 1:** Comparison of AREX_ΔST_ and AREX for bioreactor.

	AREX_ΔST_ [10^–3^ Hz]	AREX(− 3.5 ppm) [10^–3^ Hz]	Contribution of DS to AREX_ΔST_ [10^–3^ Hz]	Contribution of ssMT to AREX_ΔST_ [10^–3^ Hz]
Medium	2.8 ± 0.2	2.5 ± 0.1	0.2 ± 0.1	−
MCAs in medium	3.8 ± 0.4	3.5 ± 0.3	0.3 ± 0.1	−
Cell culture on MCAs in medium	13.9 ± 0.8	11.8 ± 0.4	1.4 ± 0.1	0.9 ± 0.2

Contribution of DS and ssMT to AREX_ΔST_ were calculated by using the Lorentzian fit result from the calculation of AREX. In Z-spectra of medium and MCAs in medium no ssMT was apparent. Thus, for these samples the Lorentzian fit constituted only of DS.

To monitor the cellular heat shock response by the rNOE CEST signal, a reliable and fast dynamic CEST technique was implemented. Chen et al.^15^ recently proposed a fast 2-point contrast metric (i.e. AREX_ΔST_, Eq. 1), which required only an offset measurement at 8 ppm and −3.5 ppm. To investigate the applicability of this contrast metric to the bioreactor system, the Z-spectra shown in Fig. 1a were analysed by comparing the rNOE signal calculated by AREX and the above-mentioned 2-point contrast metric AREX_ΔST_ (Eq. 1). For all three samples, AREX_ΔST_ resulted in an only slightly higher rNOE signal compared to the reference AREX evaluation (Table 1). The calculation of AREX required a Lorentzian fit of direct saturation (DS) and semi-solid magnetization transfer (ssMT). Based on the fit result, the residual contribution of DS and ssMT to AREX_ΔST_ was estimated (Table 1). The residual contribution of the DS and ssMT to AREX_ΔST_ was approximately 10% and 6%, respectively. Without these residual contributions AREX and AREX_ΔST_ were the same within the 95% confidence interval. Thus, the 2-point contrast metric suppressed the DS and ssMT contribution to a high degree verifying the assignment of AREX_ΔST_ to the rNOE signal. This allowed us to monitor the cellular heat shock response by fast dynamic measurements of the rNOE signal with a temporal resolution of 1 min.

### Control experiments

**Figure 2:**
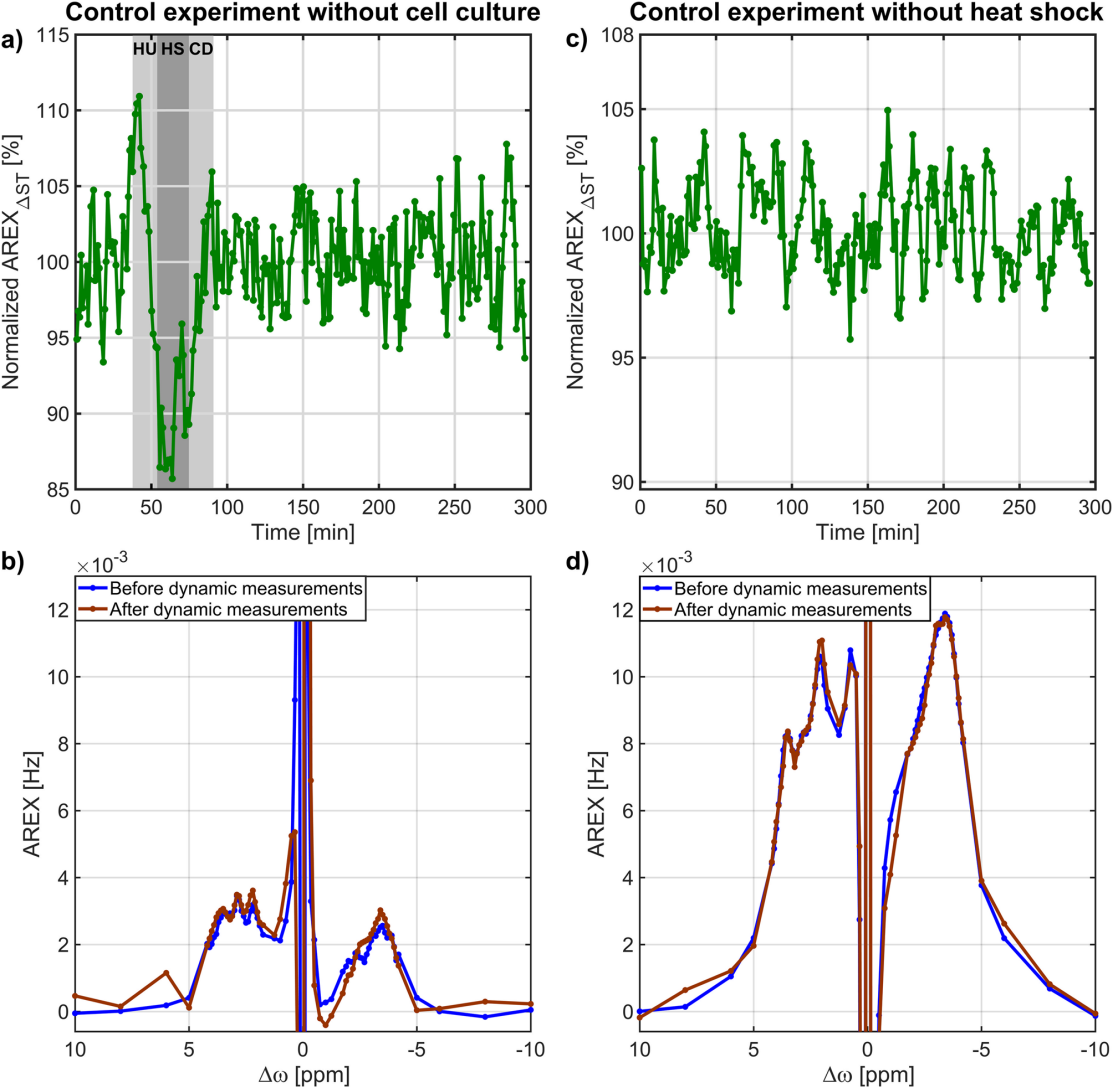
(**a**) rNOE time course of the effect of the heat shock on collagenized MCAs in medium. The dark grey shaded background indicates the heat shock (HS) of 42 °C, while the two light grey shaded backgrounds indicate the heat up (HU) and cool down (CD) to 37 °C. (**c**) Temporal stability of the rNOE signal from a cell culture during dynamic measurements but without heat shock application. The rNOE signal was constant over time, which was also confirmed by similar AREX spectra before and after dynamic measurements acquired at time 0 and 295 min respectively, as shown in (**b**) and (**d**).

The rNOE signal potentially could also be altered by the effect of the heat shock on the background rNOE signals (i.e. from medium or collagenized MCAs) or by temporal signal fluctuations, which would confound the interpretation of rNOE signal changes during heat shock experiments with cells. In Fig. 2a, the effect of a 42 °C heat shock on the rNOE background signal from the collagenized MCAs in medium is shown. The application of the heat shock had no significant impact on the dynamic course of the background rNOE signal. Only during the heat shock an initial increase in the rNOE signal and thereafter a strong reduction was observed which could be explained by a mismatch between the T_1_ and CEST measurements, or a prolonged T_1_ at 42 °C and therefore reduced levels of saturation. As the temperature cooled down to 37 °C, the rNOE signal was the same as before the heat shock and no further rNOE signal changes were observed. This was also confirmed by nearly identical AREX spectra before and after the dynamic measurements (Fig. 2b). In all heat shock experiments, the full AREX spectra labelled as before and after the dynamic measurement were acquired at time 0 and 295 min of the rNOE signal time course, respectively. The temporal stability of the rNOE signal of a cell culture during dynamic measurements but without heat shock application is presented in Fig. 2c. Again, the normalized rNOE signal showed only fluctuations around the mean value, while these fluctuations were smaller than in the dynamic measurement without a cell culture (cf. Figure 2a,c, please note the different y-axis ranges). Almost equal AREX spectra confirmed that the rNOE signal was constant during the heat shock protocol (Fig. 2d). Thus, the rNOE signal was stable over time and there was no detectable change of the background signal after the heat shock that would have originated from medium or collagenized MCAs.

### Heat shock experiments

**Figure 3:**
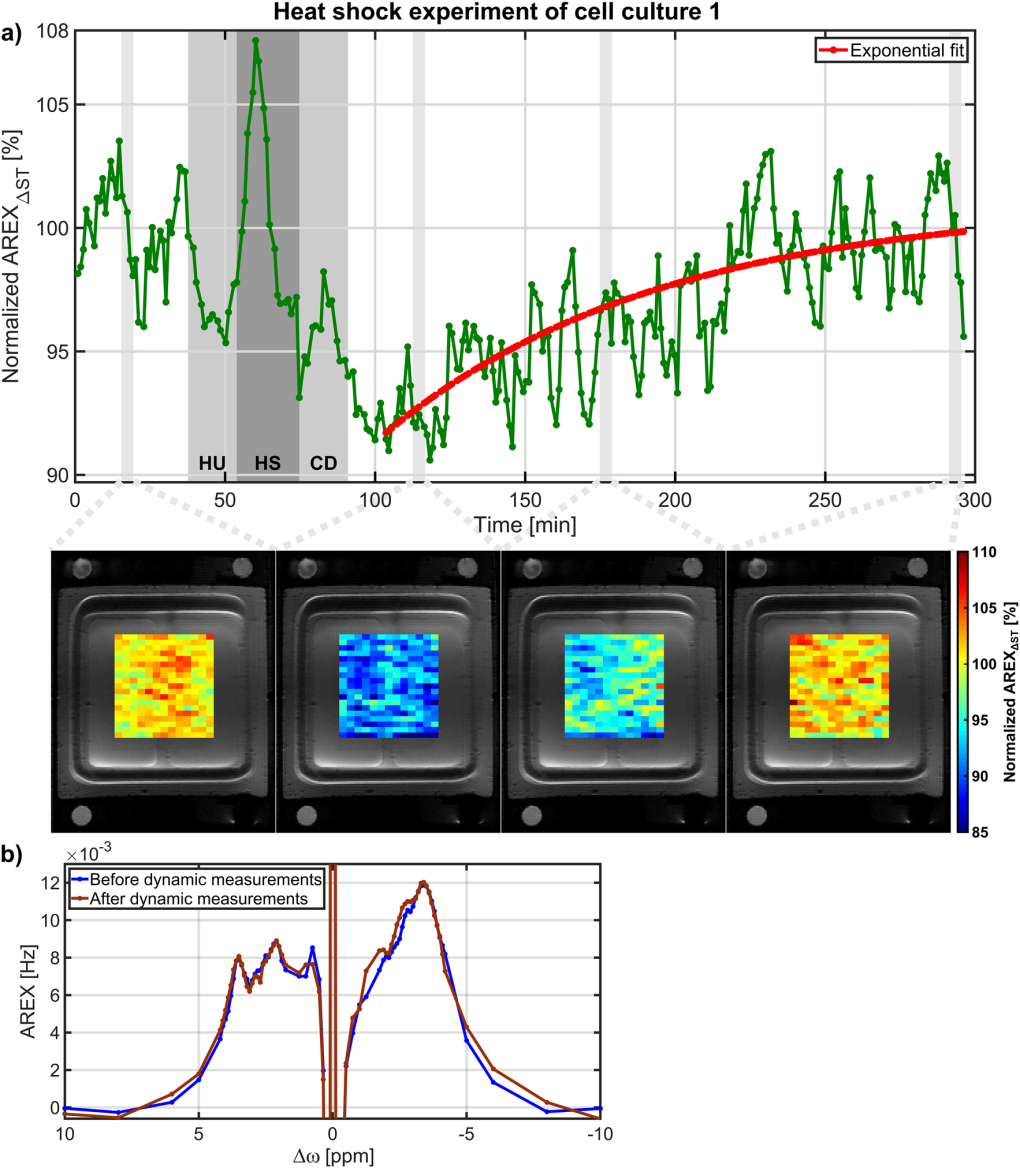
(**a**) Time course of the rNOE signal from cell culture 1 during dynamic measurements. The heat shock (HS) of 42 °C is indicated by the dark grey shaded background, while the two light grey shaded backgrounds close by indicate the heat up (HU) to 42 °C and the cool down (CD) to 37 °C. Below the time course, normalized AREX_ΔST_ images of the cell area overlaid on a ^1^H RARE image are shown. After the heat shock, a substantial rNOE signal reduction of 8.3 ± 1.1% followed by an exponential recovery with a recovery time of 100.0 ± 52.5 min was observed. An rNOE signal recovery to the initial value was also confirmed by the AREX spectra before and after dynamic measurements acquired at time 0 and 295 min, respectively, which are shown in (**b**).

**Figure 4:**
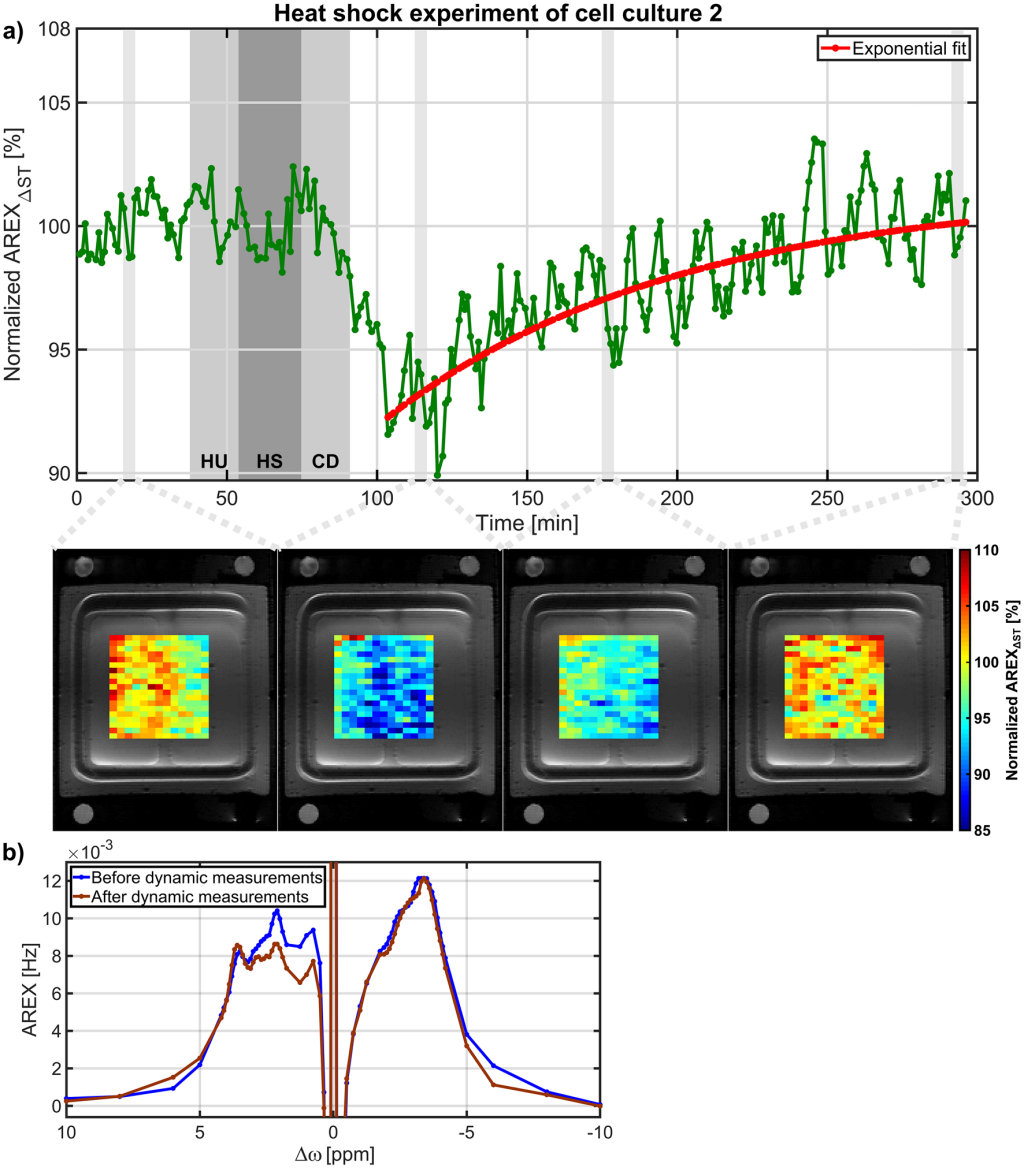
(**a**) Time course of the rNOE signal from cell culture 2 during heat shock. The dark grey shaded background indicates the heat shock (HS) of 42 °C and the two light grey shaded backgrounds close by indicate the heat up (HU) to 42 °C and the cool down (CD) to 37 °C. Below the time course, normalized AREX_ΔST_ images of the cell area overlaid on a ^1^H RARE image are shown. Similarly to the dynamic measurements of cell culture 1 (Fig. 3), the rNOE signal substantially reduced by 7.7 ± 0.8% followed by an exponential recovery with a recovery time of 98.1 ± 42.4 min. A recovery of the rNOE signal to the initial value was also confirmed by the nearly identical AREX spectra before and after dynamic measurements acquired at time 0 and 295 min, respectively, which are shown in (**b**).

In two independent experiments, the cellular heat shock response to a mild, non-lethal heat shock was monitored by dynamic measurements of the rNOE CEST signal to verify the detectability of denaturation processes on a physiologically relevant scale. The change in the rNOE signal of cell culture 1 during dynamic measurements is shown in Fig. 3a. Before the heat shock, the rNOE signal showed a small signal oscillation similarly to a previous study^24^ with the same bioreactor. After the cooling down to 37 °C, the rNOE signal substantially decreased followed by a continuous rNOE signal increase. An exponential fit of the rNOE signal recovery (Eq. 2) revealed an rNOE signal reduction of 8.3 ± 1.1% (Y_start_ = 91.7 ± 1.1%) compared to the rNOE signal before heat shock application and a recovery time of T_rec_ = 100.0 ± 52.5 min. At the end of the dynamic measurements, the rNOE signal was Y_end_ = 101.4 ± 3.9%. Consequently, the rNOE signal was within its error the same value as before heat shock application as the rNOE signal was normalized to the first 37 min prior to heat shock application. This was confirmed by nearly identical AREX spectra before and after dynamic measurements (Fig. 3b). A very similar rNOE signal response was observed with cell culture 2 during dynamic measurements (Fig. 4a). After the temperature had reached 37 °C again, a substantial reduction in the rNOE signal of 7.7 ± 0.8% (Y_start_ = 92.3 ± 0.8%) was measured, followed by an exponential-like recovery. The recovery time of T_rec_ = 98.1 ± 42.4 min was comparable to cell culture 1. The rNOE signal at the end of the dynamic measurements was Y_end_ = 101.0 ± 2.0%, which within its error nicely corresponded to the initial rNOE signal. The nearly identical rNOE signal was further confirmed by both AREX spectra before and after dynamic measurements (Fig. 4b). Additionally to the time courses, the CEST technique also allows the visualization of these rNOE signal changes in high-resolution images, see the rNOE maps below each time course in Figs. 3a and 4a. The inhomogeneity of the rNOE maps in Fig. 4a originates from inherent signal fluctuations as the noise suppression was only applied on the ROI averaged time courses.

## Discussion

The rNOE CEST signal, which can be mainly associated with mobile proteins and peptides^30^, has great potential for the investigation of diseases associated with pathological changes in protein expression and proteome structure. The rNOE signal depends on the protein content^9,17^ and the protein folding state^16–18^, whereas the dependence on the protein folding state was yet only experimentally demonstrated in protein solutions and cell lysates. Therefore, the detectability of denaturation processes and the subsequent cellular heat shock response on a physiologically relevant scale in living organisms remained to be verified experimentally. In this study, the cellular heat shock response of an organotypic 3D cell culture after a mild, non-lethal heat shock of 42 °C using an MR-compatible bioreactor system was monitored by dynamic measurements of the rNOE signal. These results aim to provide a deeper understanding of rNOE signal alterations in vivo and its capability to serve as a potential biomarker in diseases with aberrant protein folding states.

Apart from intrinsically disordered proteins^31^ (IDPs) the three-dimensional integrity of cellular proteins is crucial for their specific biological function. However, this native structure is often only marginally stable. As a consequence, elevated temperatures cause the protein unfolding, which is usually followed by aggregation due to exposed hydrophobic regions. Furthermore, a substantial number of proteins reside in a metastable state, in vivo, meaning that their concentration is higher than their solubility, making them prone to aggregation upon temperature stress^32–35^. To prevent aggregation as well as to dissolve and refold protein aggregate deposits the cellular heat shock response reacts by overexpressing molecular chaperones^36,37^. The latter are proteins which keep unfolded proteins soluble, actively refold them or dissolve aggregated protein deposits to allow their refolding^38^. Protein unfolding and aggregation caused by temperature stress can explain the observed reduction in the rNOE signal after heat shock application observed in both cell culture experiments (Figs. 3a, 4a). Notably, this rNOE signal reduction is consistent with the observed rNOE signal reduction in protein unfolding^17,18^ and aggregation^16^ experiments of protein solutions and cell lysates. However, a heat shock not only causes unfolding and aggregation of proteins, but also affects the internal organization of the cell, like reorganization of the cytoskeleton and fragmentation of organelles^37^. Therefore, the contribution of other heat shock induced cellular effects to the observed rNOE signal reduction cannot be excluded.

The exposure of cells to elevated temperatures can be lethal depending on the time and temperature of the heat shock due to the accumulation of misfolded proteins^39^. To minimize cell death, a mild, non-lethal heat shock of 42 °C for 20 min was applied in this study, as experiments of cells exposed to a similar heat shock showed that the survival rate was close to one^40–42^. However, in this case the accumulation of misfolded proteins compared to the total proteome is expected to be rather low^41–44^, which was also suggested by the rNOE signal reduction of 8.3 ± 1.1% and 7.7 ± 0.8%. In contrast, the high survival rate was of importance for the observation of the rNOE recovery, as only viable cells can refold unfolded and aggregated proteins by molecular chaperones^20,45,46^. This chaperone-induced protein refolding process after heat shock therefore increases the number of correctly folded proteins, which would be reflected in the observed rNOE signal recovery. The time from heat shock to full structural recovery of the proteome can last up to several hours dependent on the severity of the heat shock^37^. Interestingly, in hamster cells exposed to a comparable mild, non-lethal heat shock, a similar time duration to complete protein refolding was found^47^. In principle, misfolded proteins can also be degraded and newly synthesized^20^. However, a recent study^43^ in budding yeast cells exposed to a mild, non-lethal heat shock showed that even severely aggregated proteins were refolded instead of degraded. The recovery of the rNOE signal to the initial value is consistent with the disaggregation of proteins without degradation and with the expected high survival rate of cells.

For the comparison of the heat shock experiments from both cell cultures, the rNOE signal recovery was fitted by an exponential function (Eq. 2). Notably, heat shock experiments in eukaryotic cells monitored by luciferase activity^41,47–50^ or by nuclear protein content^40,42,45,46,51^ showed a similar exponential-like recovery after the exposure to heat. However, to the best of our knowledge a quantification of the heat shock recovery has not been performed so far. Based on literature analysis and the results of the heat shock experiments in this study, quantification of the heat shock recovery by an exponential function seemed to be a good representation of the available data and should facilitate the comparability of heat shock experiments.

To rule out further causes for the observed rNOE signal recovery, the effect of a heat shock on the background rNOE signal from medium and collagenized MCAs and the temporal stability of the rNOE signal without heat shock application was investigated (Fig. 2). However, rNOE signal changes that occur during the application of the heat shock should be interpreted with care, as T_1_ strongly varies as a function of temperature^52^ leading to different levels of saturation^29,53^. In contrast, explaining the observed rNOE signal recovery simply by T_1_ changes (Eq. 1) could be ruled out, as in all heat shock experiments a constant T_1_ value after cooling to 37 °C until the end of the experiment was observed (Fig. 6a). Furthermore, the T_1_ value after the heat shock reached the same level as prior to heat shock application. This was also confirmed by the calculation of the rNOE signal by alternative metrics without T_1_ correction (i.e. MTR_Rex_^54^ or ΔST^15^, Supplementary Eq. 1, 2 and Supplementary Figures S1,S2) which showed a similar recovery of the rNOE signal with a comparable T_rec_ (Supplementary Table S1). Thus, the effect of a T_1_ recovery as an explanation for the observed rNOE signal recovery could be ruled out.

The applied 2-point contrast metric, proposed by Chen et al.^15^, to achieve fast and reliable CEST imaging, sufficiently suppressed confounding contributions to the Z-spectrum (i.e. from DS and ssMT) to selectively investigate changes in the rNOE signal. The 2-point contrast metric suppresses DS and ssMT by assuming that these contributions are only marginally different between the two offset frequencies 8 and −3.5 ppm^15^. Although, this method was developed at an ultrahigh magnetic field strength of 11.7 T, we were also able to confirm the applicability of AREX_ΔST_ in the bioreactor system at 9.4 T (Table 1). Nonetheless, the aggregation of proteins during the heat shock experiments could have caused an increase in ssMT^15,16^, which would alter the residual ssMT contribution. However, the amount of residual ssMT contribution of approximately 6.5% is not enough to explain the observed average change in rNOE signal upon heat shock of about 8.0 ± 0.4%. With the used setup, also field inhomogeneities across the cell culture do not have a large impact on the rNOE signal calculation by the 2-point metric. Regarding B_0_, the inhomogeneity was smaller than ± 0.2 ppm and changed only marginally by ± 0.02 ppm during dynamic measurements, which is negligible in comparison to the observed spectral widths of the peaks in the acquired Z-spectra (Fig. 1). During the dynamic measurements, a possible change in the water resonance frequency was prevented by setting the global frequency for each 2-point and saturation recovery measurement. With respect to B_1_^+^, inhomogeneities were also negligible using a quadrature ^1^H birdcage coil with an average B_1_^+^ deviation smaller than 2% across the entire cell culture. In addition, B_1_^+^ values were set consistently to 0.8 µT between all measurements. With the presented bioreactor CEST MRI setup at hand, besides heat shock experiments, in the future one could also investigate CEST signal changes upon disease progression or treatment-related contrast changes in an isolated manner to identify potential new biomarkers. In doing so, ultrafast CEST techniques^55–57^, which allow the acquisition of the full Z-spectrum with a high temporal resolution during the intervention, would greatly assist the study of the involved mechanisms.

## Conclusion

The detectability of protein denaturation processes on a physiologically relevant scale in living cells by CEST MRI was demonstrated using the rNOE signal. The application of a mild, non-lethal heat shock to organotypic 3D cell cultures located in an MR-compatible bioreactor system combined with reliable and fast high-resolution CEST imaging allowed us to observe the cellular heat shock response. The application of a mild, non-lethal heat shock resulted in a substantial decrease of the rNOE signal followed by a continuous rNOE signal recovery. As the CEST signals had been shown to mainly originate from the 3D cell culture, these rNOE signal changes can be explained by denaturation of proteins during heat shock application and refolding of misfolded proteins by molecular chaperones. These results demonstrate that the protein folding state, beside changes in concentration, is a relevant physiological parameter for the interpretation of CEST signal changes. The dependence on the protein folding state can be of interest as a non-invasive diagnostic tool in diseases which are associated with pathologic changes in protein expression and proteome structure, like cancer and neurodegenerative diseases.

## Material and methods

Data acquisition was performed on a 9.4 T preclinical MRI (Bruker Biospec 94/20, Ettlingen, Germany). A Bruker quadrature ^1^H birdcage coil, which achieves a homogenous RF excitation, was combined with a Bruker rat receiver surface array for higher signal sensitivity.

### MR-compatible microcavity array-based bioreactor system

**Figure 5:**
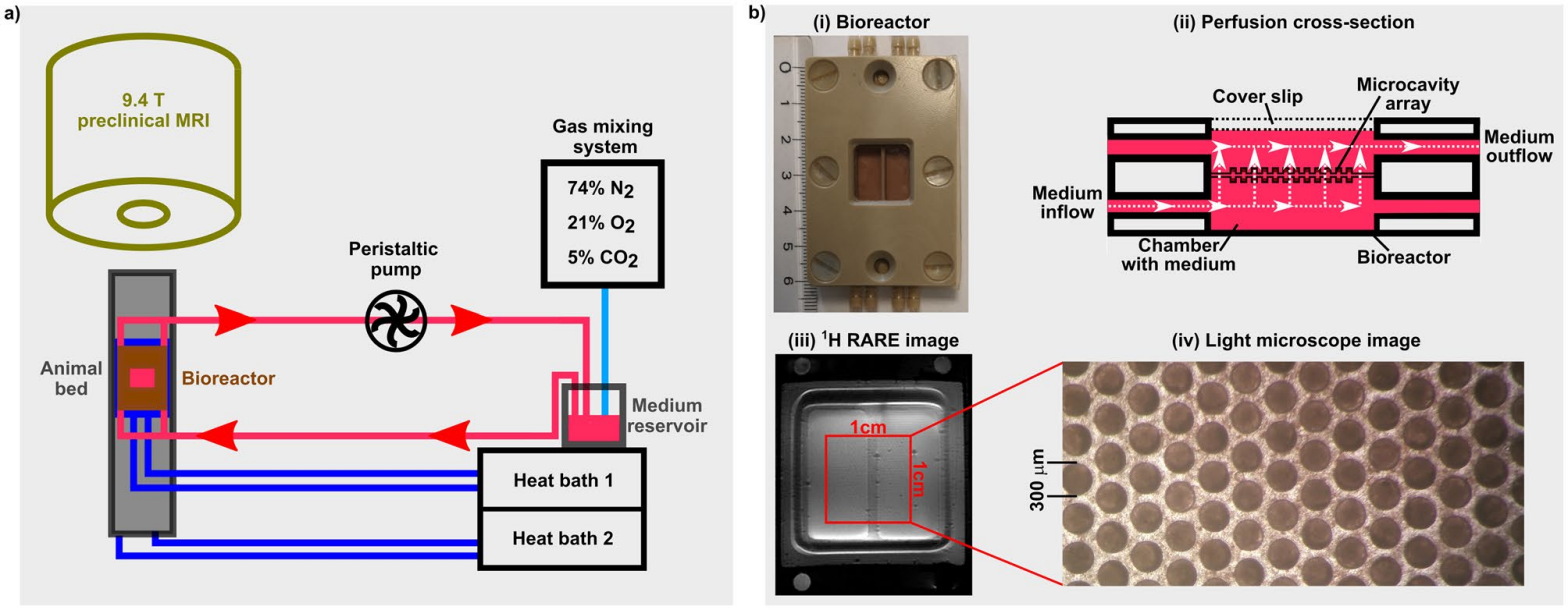
(**a**) Graphical sketch of the bioreactor setup. A peristaltic pump supplied cells at 400 µl/min with fresh medium, which was aerated by a gas mixing station. A temperature of 37 °C inside the bioreactor was achieved by heating the animal bead and the tubes prior to the bioreactor by the first heat bath. To achieve a heat shock of 42 °C, a second heat bath was added to the bioreactor setup. (**b**) (i) Dimension of bioreactor, (ii) bioreactor cross-section with perfusion of the medium, (iii) ^1^H RARE image of the bioreactor loaded with two MCAs and (iv) light microscope image of one MCA are shown. Fresh medium entered the bioreactor at the bottom, which was then perfused through the pores of the MCAs and subsequently left the bioreactor on top. The size of the cavity area is 1 × 1 cm^2^, while each cavity has a depth and diameter of 300 µm.

An MR-compatible bioreactor enables the non-invasive measurement of cellular responses in living cells under a precisely controlled environment. This bioreactor contains an organotypic 3D culture on a MCA which consists of 634 cavities on an area of 1 × 1 cm^2^. Each cavity has a depth and a diameter of 300 µm. The microcavity array, manufactured from polycarbonate, was surface modified by physisorption of callagen I from rat tail^58^. For this, after a hydrophilization step with an isopropanol series, a collagen I-solution (0.24 mg/ml) was pipetted onto the array and incubated at 37 °C for 1 h. After this, a drop of 150 µl medium containing 8–9 × 10^6^ hepatocellular carcinoma cells of line HepG2 (ATCC HB-8065, Manassas, USA) was placed on the collagen-coated MCA. Preparation of HepG2 cells was performed according to previous reports^22,59,60^. The MCA with cells was placed inside an incubator for initial cultivation before transferring the MCA into the bioreactor. Two MCAs with the upper array placed upside down were used. Inside the bioreactor, cells were actively perfused by a peristaltic pump with 400 µl/min medium through pores within the MCA (Fig. 5). The medium consisted of minimum essential medium (MEM) with 10% fetal bovine serum, 1% glutamax, 1% non-essential amino acids, 1% sodium pyruvate, 1% penicilin-streptomycin and 0.1% phenol red. The medium contained 3–4 mg/ml proteins while 16–18·10^6^ HepG2 cells resulted in a protein concentration of 16–18 mg/ml. The medium was aerated with 74% N_2_, 21% O_2_ and 5% CO_2_ provided by a gas mixing system (Fig. 5). The bioreactor was placed on a heated animal bed and maintained at a temperature of 37 °C which was controlled with an infrared thermometer (Voltcraft IR-SCAN-350RH, Hirschau, Germany).

For each measurement, the bioreactor was actively perfused under normoxic conditions at 37 °C.

**Figure 6:**
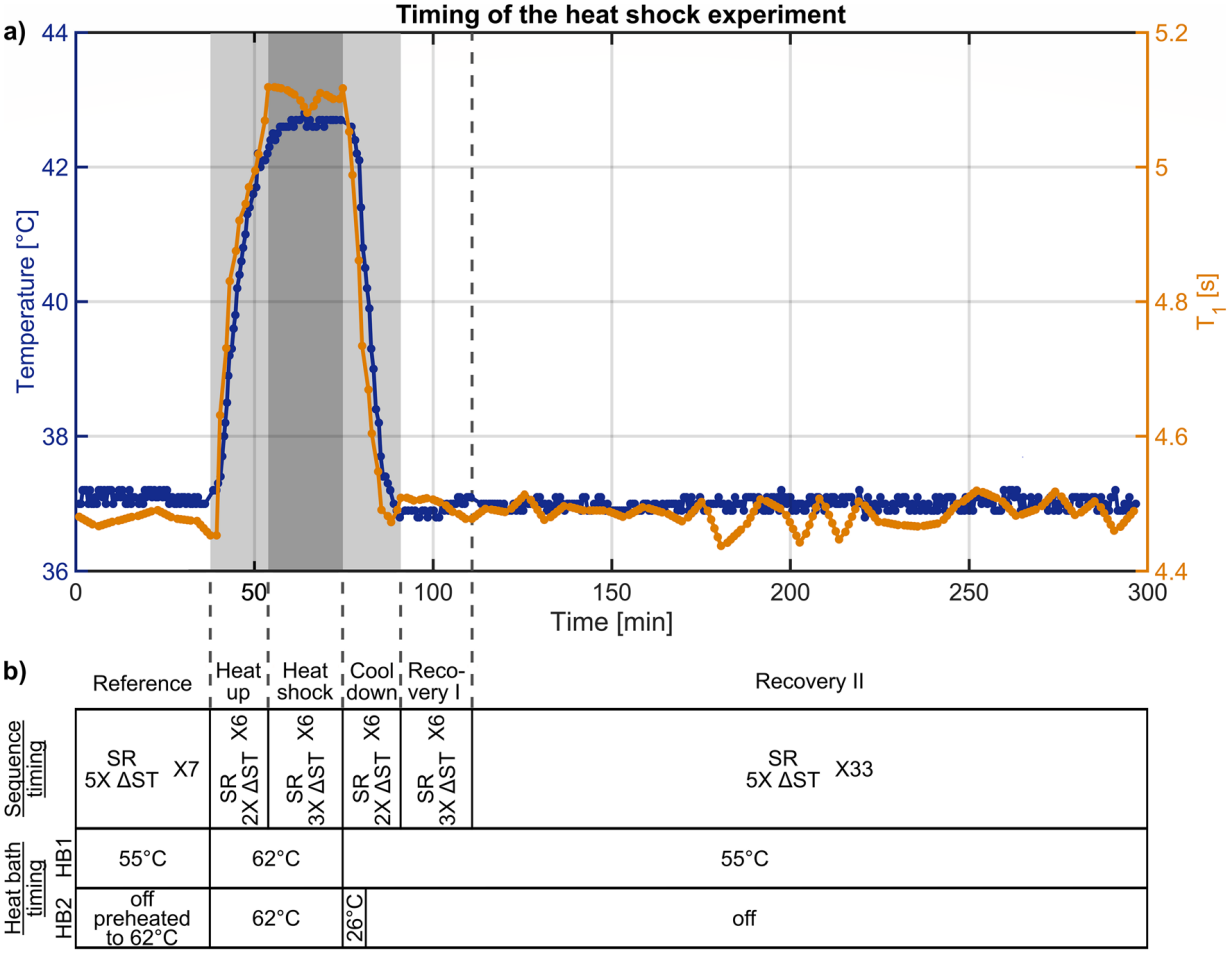
(**a**) Temperature and T_1_ time course during heat shock of 42 °C. Temperature was assessed by an infrared thermometer by placing the bioreactor with collagenized MCAs in medium on the animal bed in front of the MRI. The T_1_ time course is from the heat shock experiment of cell culture 1. The heating up to 42 °C and the cooling down to 37 °C took approximately 15 min, while cells were exposed to a heat shock of 42 °C for 20 min. The change in T_1_ correlates well with the observed temperature change. After the 15 min cool down, the T_1_ value was constant showing that the rNOE signal changes could not be explained by a recovery of T_1_. (**b**) Sequence and heat bath timing of the heat shock protocol. T_1_ was finer sampled around the heat shock of 42 °C. For a fast heating up to 42 °C, the second heat bath was preheated, while a rapid cooling to 37 °C was achieved by addition of ice-cold water to both heat baths.

The 3D cell cultures were exposed to a mild, non-lethal heat shock of 42 °C for 20 min inside this bioreactor system. To achieve this heat shock, the bioreactor setup was extended by a second water bath. Prior to the MRI measurements, the parameters and switching times for both water baths were determined by the infrared thermometer (Fig. 6b). Therefore, a heat shock experiment with the bioreactor containing two collagenized MCAs placed on the animal bed in front of the MRI was performed. After the heat shock, a rapid cooling to 37 °C was achieved by addition of ice-cold water to both water baths. The time to heat up to 42 °C and to cool down to 37 °C was in both cases 15 min (Fig. 6a).

### Spectroscopic CEST Imaging

A continuous wave block pulse of RF amplitude B_1_ = 0.8 µT and length t_sat_ = 10 s followed by crusher gradients and a RARE readout was used. The RARE readout was centric reordered with following parameters: T_E_ = 2.86 ms, RARE factor 32, imaging matrix = 70 × 50, slice thickness 2 cm and FoV = 35 × 35 cm^2^. Before and after dynamic CEST measurements, a full Z-spectrum Z(Δω) = M_sat_(Δω)/M_0_(Δω) was sampled at 93 non-equidistant frequency offsets between Δω =  ± 200 ppm. To minimize signal fluctuations, an equilibrium magnetization M_0_(Δω) was acquired before and after acquisition of M_sat_(Δω). M_0_(Δω) was linearly interpolated to obtain an individual M_0_(Δω) for each saturation frequency. T_1_ was measured with a saturation recovery (SR) sequence. Water signal saturation was achieved by three adiabatic hyperbolic secant RF pulses with crusher gradients in between the RF pulses. Signal was acquired by a linear ordered, RARE readout (i.e. T_E_ = 45.76 ms) with identical imaging parameters to the CEST measurements. 30 non-equidistant spaced recovery times between 4.8 ms and 25 s were measured.

### Dynamic CEST imaging

For a high temporal resolution of 1 min, dynamic CEST measurements interleaved with SR measurements were performed from 37 min before the heat shock until 200 min after the heat shock (Fig. 6b). Dynamic CEST measurements consisted of a two offset CEST measurement at 8 ppm and −3.5 ppm, which was recently proposed by Chen et al.^15^. For dynamic measurements, six recovery times [0.0048 0.1 3.0 5.0 10.0 17.5] s were sampled for the SR measurement. The other sequence parameters were identical to the described parameters above. The T_1_ sampling was adapted for a finer sampling around the heat shock and a sparser sampling before and after the heat shock (Fig. 6b). T_1_ values were linearly interpolated to obtain one T_1_ value for each of the two offset CEST measurement. Prior to each CEST or SR measurement the water frequency was set to correct for frequency drifts over time.

### CEST Evaluation

The contributions of DS, ssMT and T_1_ relaxation to Z-spectra were removed by calculation of AREX(Δω) = 1/T_1_ (1/Z_lab_(Δω)−1/Z_ref_(Δω))^29^. The measured Z-values are labelled as Z_lab_(Δω), while Z_ref_(Δω) represents an estimation of DS and ssMT by a multi-parametric fit: Z_ref_(Δω) = 1 – (L_DS_(Δω) + L_ssMT_(Δω)). L_DS_(Δω) and L_ssMT_(Δω) are Lorentzian-shaped functions. For the multi-parametric fit, only data points in the spectral range of [± 200, ± 8] ppm and [−0.75, 0.75] ppm were used to avoid contributions from selective saturation transfer effects. ssMT was not apparent in Z-spectra of medium and Z-spectra of collagenized MCAs in medium. Thus, in those samples Z_ref_(Δω) consisted of only one Lorentzian-shaped function for DS.

The proposed 2-point contrast metric by Chen et al.^15^ was adapted to the AREX metric:

1 $${\text{AREX}}_{{\Delta {\text{ST}}}} \left( { - {3}.{5}\,{\text{ppm}}} \right) = {1}/{\text{T}}_{{1}} \left( {{1}/{\text{Z}}\left( { - {3}.{5}\,{\text{ppm}}} \right){-} {1}/{\text{Z}}\left( {{8}\,{\text{ppm}}} \right)} \right)$$

The quantity AREX_ΔST_(−3.5 ppm) describes the approximated rNOE signal at −3.5 ppm from which the contribution of DS and ssMT was removed by the offset measurement at 8 ppm. The applicability of AREX_ΔST_ to the bioreactor system was verified by comparison of AREX_ΔST_(−3.5 ppm) with AREX(−3.5 ppm) (Table 1). Residual contributions from DS and ssMT to AREX_ΔST_(−3.5 ppm) were calculated from the estimated Lorentzian fit of DS and ssMT for Z_ref_(Δω). For dynamic measurements, AREX_ΔST_(−3.5 ppm) was normalized pixel-wise to the first 37 min prior to the heat shock. Then the ROI averaged AREX_ΔST_(−3.5 ppm) was calculated and the time course was convolved with a block pulse of width four for noise suppression.

### Quantification of heat shock recovery

The cellular heat shock response resulted in a continuous increase in the rNOE signal comparable to an exponential function. To quantify the heat shock response, the time course from 100 min, which corresponds to the time immediately after the temperature returned to 37 °C, until the end was fitted by:

2 $${\text{Y}}\left( {\text{t}} \right) = \left( {{\text{Y}}_{{{\text{start}}}} - {\text{Y}}_{{{\text{end}}}} } \right)\,{\exp}\left( { - {\text{t}}/{\text{T}}_{{{\text{rec}}}} } \right) + {\text{Y}}_{{{\text{end}}}}$$

where Y(t) was the normalized AREX_ΔST_(−3.5 ppm); Y_start_ is the minimum value; Y_end_ is the maximum value and T_rec_ is the recovery time. The standard deviation of all fit parameters corresponds to the 95% confidence interval of the Levenberg–Marquardt fit.

## Supplementary information

Supplementary information

## Data Availability

The datasets generated during and/or analysed during the current study are available from the corresponding author upon request.
