# Supplementary material for: The cellular heat shock response monitored by chemical exchange saturation transfer MRI
Source: Sci Rep. 2020 Jul 6;10:11118. doi: 10.1038/s41598-020-68022-1 (PMC7338423; doi:10.1038/s41598-020-68022-1)
Supplement: Supplementary file 1 — Supplementary information [file 41598_2020_68022_MOESM1_ESM.docx]

**Supplementary Information**

**The cellular heat shock response monitored by chemical exchange saturation transfer MRI**

*Dennis Kleimaier, Steffen Goerke, Cordula Nies, Moritz Zaiss, Patrick Kunz, Peter Bachert, Mark E. Ladd, Eric Gottwald, and Lothar R. Schad*

**Alternative CEST Evaluation**

The two offset CEST measurement can also be evaluated by the saturation transfer difference ΔST[^1^](#_ENREF_1) or by the spillover corrected magnetization transfer ratio MTR_Rex_[^2^](#_ENREF_2):

ΔST(−3.5 ppm) = Z(8 ppm) – Z(−3.5 ppm)) (S1)

MTR_RexΔST_(−3.5 ppm) = 1/Z(−3.5 ppm) – 1/Z(8 ppm) (S2)

The quantities ΔST(−3.5 ppm) and MTR_RexΔST_(−3.5 ppm) describe the approximated rNOE signal at −3.5 ppm from which the contribution of DS and ssMT was removed by the offset measurement at 8 ppm.

The time course of the rNOE signal calculated by ΔST and MTR_RexΔST_ are presented on **Supplementary Fig. S1 and S2**. The rNOE signal calculated by any of the three metrics showed a substantial reduction of the rNOE signal after heat shock followed by an rNOE signal recovery. Analysis of all rNOE signal recoveries by **Equation 2** yielded the same values within the 95% confidence intervals for Y_start_, Y_end_ and T_Rec_ (**Supplementary Table S1**). Thus, the effect of a T_1_ recovery as an explanation for the observed rNOE signal recovery could be ruled out.


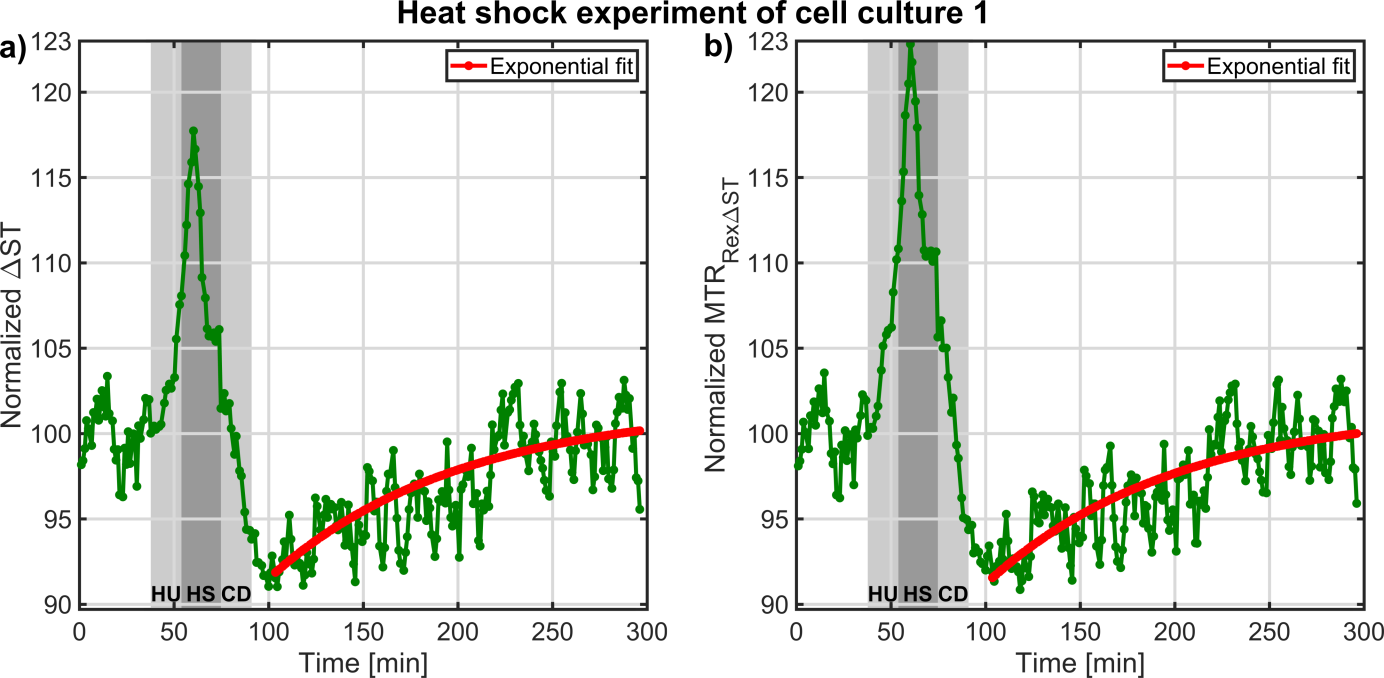
Supplementary Figure S1: a) Time course of the rNOE signal, calculated by Supplementary Equation S1, from cell culture 1 during heat shock. The dark grey shaded background indicates the heat shock (HS) of 42°C, while the two light grey shaded backgrounds indicate the heat up (HU) and cool down (CD) to 37°C. b) Time course of the rNOE signal, calculated by Supplementary Equation S2, from cell culture 1 during heat shock.


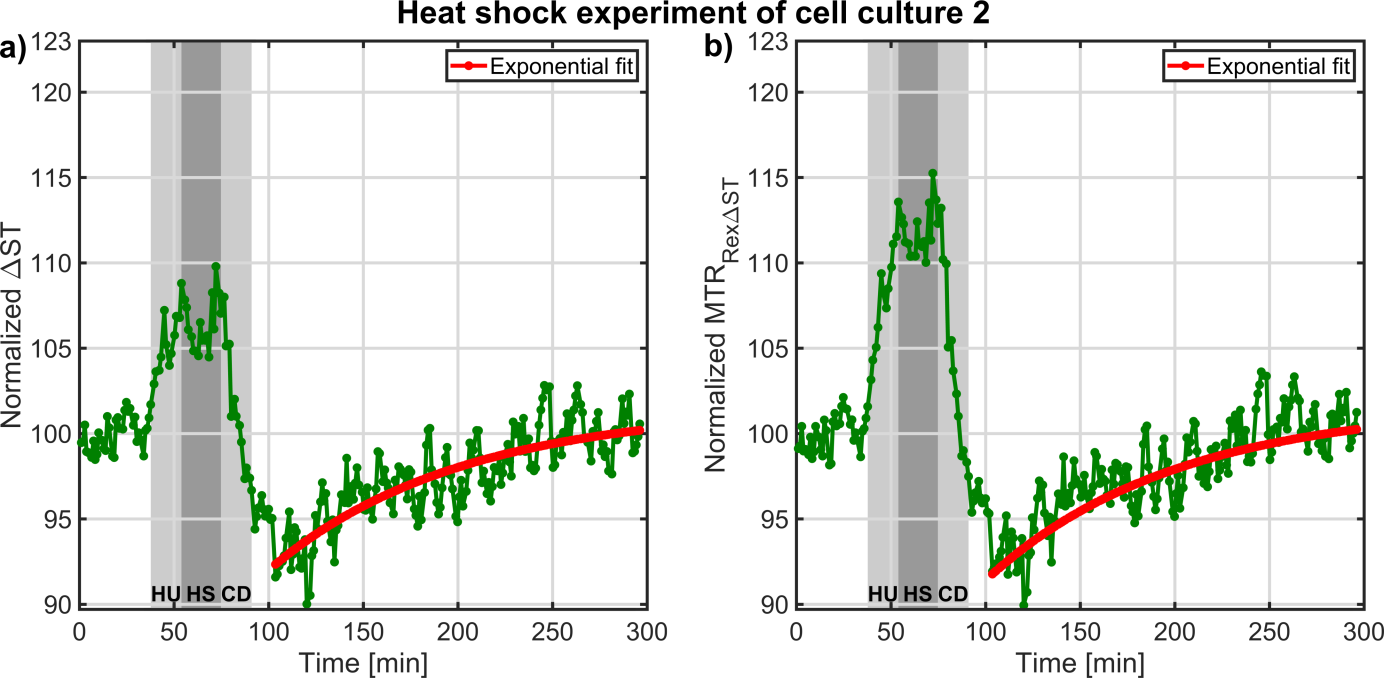
**Supplementary Figure S2**: a) Time course of the rNOE signal, calculated by **Supplementary Equation S1**, from cell culture 2 during heat shock. The dark grey shaded background indicates the heat shock (HS) of 42°C, while the two light grey shaded backgrounds indicate the heat up (HU) and cool down (CD) to 37°C. b) Time course of the rNOE signal, calculated by **Supplementary Equation S2**, from cell culture 2 during heat shock.

Supplementary Table S1: Exponential fit of the cellular heat shock response

|  | Cell culture 1 | | | Cell culture 2 | | |
| --- | --- | --- | --- | --- | --- | --- |
|  | AREX_ΔST_ | MTR_Rex_ | ΔST | AREX_ΔST_ | MTR_RexΔST_ | ΔST |
| Y_start_ | 91.7 ± 1.1% | 91.6 ± 1.1% | 91.9 ± 1.1% | 92.3 ± 0.8% | 91.8 ± 0.8% | 92.3 ± 0.8% |
| Y_end_ | 101.4 ± 3.9% | 101.5 ± 2.2% | 101.6 ± 2.3% | 101.0 ± 2.0% | 101.7 ± 2.0% | 101.5 ± 1.8% |
| T_Rec_ | 100.0 ± 52.5 min | 100.0 ± 53.1 min | 100.0 ± 54.9 min | 98.1 ± 42.4 min | 100.0 ± 40.1 min | 100.0 ± 43.2 min |

Exponential function is presented by Equation 2. “Y_start_” is the minimum value, “Y_end_” is the maximum value and “T_Rec_” is the recovery time.

**Supplementary References**

1 Chen, L. *et al.* Protein aggregation linked to Alzheimer's disease revealed by saturation transfer MRI. *Neuroimage* **188**, 380-390, doi:10.1016/j.neuroimage.2018.12.018 (2019).

2 Zaiss, M. & Bachert, P. Exchange-dependent relaxation in the rotating frame for slow and intermediate exchange -- modeling off-resonant spin-lock and chemical exchange saturation transfer. *NMR Biomed* **26**, 507-518, doi:10.1002/nbm.2887 (2013).
